# The application of epiphenotyping approaches to DNA methylation array studies of the human placenta

**DOI:** 10.1186/s13072-023-00507-5

**Published:** 2023-10-04

**Authors:** A. Khan, A. M. Inkster, M. S. Peñaherrera, S. King, S. Kildea, T. F. Oberlander, D. M. Olson, C. Vaillancourt, U. Brain, E. O. Beraldo, A. G. Beristain, V. L. Clifton, G. F. Del Gobbo, W. L. Lam, G. A. S. Metz, J. W. Y. Ng, E. M. Price, J. M. Schuetz, V. Yuan, É. Portales-Casamar, W. P. Robinson

**Affiliations:** 1 BC Children’s Hospital Research Institute (BCCHR), 950 W 28th Ave, Vancouver, BC V5Z 4H4 Canada; 2 Department of Medical Genetics, University of British Columbia, Vancouver, BC V6H 3N1 Canada; 3 Department of Medical Biophysics, University of Toronto, Toronto, ON M5G 1L7 Canada; 4 Princess Margaret Cancer Center, Toronto, ON M5G 2C4 Canada; 5 Department of Psychiatry, McGill University, Montreal, QC H3A 1A1 Canada; 6 Psychosocial Research Division, Douglas Hospital Research Centre, Montreal, QC H4H 1R3 Canada; 7 Mater Research Institute, University of Queensland, Brisbane, QLD 4101 Australia; 8 Molly Wardaguga Research Centre, Charles Darwin University, Brisbane, QLD 4000 Australia; 9 School of Population and Public Health, University of British Columbia, Vancouver, BC V6T 1Z3 Canada; 10 Department of Pediatrics, University of British Columbia, Vancouver, BC V6H 3V4 Canada; 11 Department of Obstetrics and Gynecology, University of Alberta, 220 HMRC, Edmonton, AB T6G 2S2 Canada; 12 Centre Armand Frappier Santé Biotechnologie - INRS and University of Quebec Intersectorial Health Research Network, Laval, QC H7V 1B7 Canada; 13 Department of Obstetrics & Gynecology, University of British Columbia, Vancouver, BC V6T 1Z3 Canada; 14 Faculty of Medicine, The University of Queensland, Herston, QLD 4006 Australia; 15 Children’s Hospital of Eastern Ontario Research Institute, University of Ottawa, Ottawa, ON K1H 5B2 Canada; 16 British Columbia Cancer Research Centre, Vancouver, BC V5Z 1L3 Canada; 17 Canadian Centre for Behavioural Neuroscience, Department of Neuroscience, University of Lethbridge, Lethbridge, AB T1K 3M4 Canada; 18 Faculty of Medicine, University of Calgary, Calgary, AB T2N 4N1 Canada; 19 Centre de Recherche du CHU Sainte-Justine, 3175 Côte-Sainte-Catherine Road, Montréal, QC H3T 1C5 Canada

**Keywords:** DNA methylation, Placenta, Epiphenotyping, Epigenetics, Epigenetic age, Gestational age, Cell composition, Ancestry, PlaNET R package

## Abstract

**Background:**

Genome-wide DNA methylation (DNAme) profiling of the placenta with Illumina Infinium Methylation bead arrays is often used to explore the connections between in utero exposures, placental pathology, and fetal development. However, many technical and biological factors can lead to signals of DNAme variation between samples and between cohorts, and understanding and accounting for these factors is essential to ensure meaningful and replicable data analysis. Recently, “epiphenotyping” approaches have been developed whereby DNAme data can be used to impute information about phenotypic variables such as gestational age, sex, cell composition, and ancestry. These epiphenotypes offer avenues to compare phenotypic data across cohorts, and to understand how phenotypic variables relate to DNAme variability. However, the relationships between placental epiphenotyping variables and other technical and biological variables, and their application to downstream epigenome analyses, have not been well studied.

**Results:**

Using DNAme data from 204 placentas across three cohorts, we applied the PlaNET R package to estimate epiphenotypes gestational age, ancestry, and cell composition in these samples. PlaNET ancestry estimates were highly correlated with independent polymorphic ancestry-informative markers, and epigenetic gestational age, on average, was estimated within 4 days of reported gestational age, underscoring the accuracy of these tools. Cell composition estimates varied both within and between cohorts, as well as over very long placental processing times. Interestingly, the ratio of cytotrophoblast to syncytiotrophoblast proportion decreased with increasing gestational age, and differed slightly by both maternal ethnicity (lower in white vs. non-white) and genetic ancestry (lower in higher probability European ancestry). The cohort of origin and cytotrophoblast proportion were the largest drivers of DNAme variation in this dataset, based on their associations with the first principal component.

**Conclusions:**

This work confirms that cohort, array (technical) batch, cell type proportion, self-reported ethnicity, genetic ancestry, and biological sex are important variables to consider in any analyses of Illumina DNAme data. We further demonstrate the specific utility of epiphenotyping tools developed for use with placental DNAme data, and show that these variables (i) provide an independent check of clinically obtained data and (ii) provide a robust approach to compare variables across different datasets. Finally, we present a general framework for the processing and analysis of placental DNAme data, integrating the epiphenotype variables discussed here.

**Supplementary Information:**

The online version contains supplementary material available at 10.1186/s13072-023-00507-5.

## Background

The placenta is an organ derived from cells of the conceptus, and is genetically identical to the fetus. The placenta is essential for fetal growth and development, and plays an important role in mediating maternal exposures that may influence newborn and child health. To better understand these roles of the placenta, genome-wide DNA methylation (DNAme) profiling has been widely applied, often using Illumina Infinium Methylation bead arrays. Alterations in placental DNAme have been reported in association with maternal exposures such as smoking [1, 2], gestational diabetes, and obesity [3–5], as well as in association with perinatal complications such as preeclampsia, chorioamnionitis, and low birthweight [6–11]. In some cases, these effects are intersectional: for example, smoking-associated changes in placental DNAme may be confounded, or in some cases mediated, by lower birth weight [1, 2, 12], although other lifestyle and exposure factors can complicate interpretation of these data. Despite the range of studies conducted in placenta, replication analyses of epigenome-wide association studies (EWAS) in independent populations are less common. Even in the context of early-onset preeclampsia, which is a condition associated with widespread alterations in DNAme of large effect size, reported findings are often inconsistent in independent datasets [7, 13, 14].

Part of the issue underlying incomplete replication between studies is inter-dataset heterogeneity. Prior to performing epigenome-wide analysis, it is important to understand and account for the factors driving variability in each DNAme dataset. Relevant technical factors may include differences in sample processing techniques, batch effects, and poor data quality control, which can all lead to false positive EWAS results, or low signal-to-noise ratios [6]. Biological factors that may confound analyses include differences in bulk tissue sample cell composition [15], sex [16], and gestational or chronological age [17, 18]. In addition, the ethnicity and/or genetic ancestry of subjects are known confounders in EWAS studies [19–21], and many regions of high DNAme variability across individuals are influenced by genetic variation [22, 23].

Data interpretation and replicability across studies from diverse populations and using different sample collection methods can be improved if we can better assess and account for key variables and systematic differences between datasets. “Epiphenotyping” approaches have been developed whereby the (epigenetic) DNAme data are used to extract information about phenotypic variables, such as age, sex, or cell composition [24–28]. While such approaches are increasingly used in DNAme profiling of blood and other tissues, they have not been routinely applied to the DNAme analysis of placental tissue, in part as the placenta has a very unique DNAme profile that affects the performance of previously developed tools.

Recently, epiphenotyping tools have been developed to estimate placental cell composition, ancestry, and epigenetic age from DNAme data. Since the development of these placental-specific epiphenotyping tools, and their implementation in the PlaNET R package [29], the associations between placental epiphenotype variables, and with other technical and biological variables, have not been fully characterized. This study seeks to evaluate the placental epiphenotyping tools that have recently been made available, and to test the value of their integration into the processing and analysis of placental DNAme data.

The placental epiphenotypes investigated here include: (1) a placental cell-specific deconvolution method developed to estimate major cell types in bulk tissue DNAme data [15], which can be used to assess and account for sampling variation between and within datasets, and to identify cell composition changes underlying DNAme differences between exposure groups [30–32]. (2) An approach to estimate genetic ancestry of the placenta as a continuous variable directly from the DNAme data, as ancestry may not be well captured by self-reported parental ethnicity [33]. The PlaNET approach to estimating ancestry was found to improve replication between EWAS studies, and to outperform PCA-based approaches that work well in other tissues, such as the Barfield et al. [34] or EPISTRUCTURE [35] methods [33], underscoring the need to develop placental-specific epiphenotype algorithms. (3) An epigenetic clock for estimation of gestational age at birth, which is sometimes missing or inaccurately recorded in clinical records, may provide researchers with a way to estimate missing data, to standardize measures of gestational age across studies (which can be unreliable without good clinical records), and to study placental epigenetic age acceleration [18].

In this study, we use three cohorts of placental samples (chorionic villi sampled from the fetal-facing side) to assess factors contributing to within- and between-cohort variation in placental DNAme data. We specifically apply the PlaNET R package to estimate gestational age, ancestry, and cell composition epiphenotype variables, and we evaluate the utility of these epiphenotyping approaches, assess inter-cohort differences, and examine their relationships to technical and biological variables. In addition, we explore how technical and biological variables common to placental DNAme studies are related to each other, and to the imputed epiphenotype variables. Finally, based on these extensive studies, we provide a set of recommendations for the use of these epiphenotyping tools in placental EWAS.

## Results

### Cohort characteristics

In this study, we use DNAme data from 204 placentas across three independent cohorts to investigate the relationships between placental epiphenotype variables computed with the R package PlaNET, and other technical and biological characteristics associated with these samples. The placentas from the V-NORM and V-SSRI cohorts were obtained and sampled in one processing lab located in Vancouver, Canada. The QF2011 cohort placentas were collected and sampled in Brisbane, Australia, and subsequently snap-frozen and shipped to Montreal, Canada for DNA extractions. Samples from all three cohorts were assayed on the Illumina Infinium MethylationEPIC (850K) array at one center in Vancouver, Canada, and were randomized during array processing for key variables including cohort, SSRI and flood-related stress exposure, and infant sex. The samples from all cohorts were run on the EPIC array in three technical “batches”, with batch referring to all stages of array processing from bisulfite conversion, to array hybridization and staining, to scanning. More details are presented in Table 1 and in the Methods section. Maps of all samples included in the three array batches and their randomization characteristics are presented in Additional file 1: Fig. S1. Key technical and biological variables used in this study are reported in Table 1.

**Table 1:** Summary of key biological and technical variables for each cohort. SD refers to standard deviation, QC refers to quality control

	V-NORM	V-SSRI	QF2011	*p* value*
*n*	35	64	105	
Biological variables				
Gestational age at birth (mean weeks (SD))	39.0 (1.1)	39.5 (1.3)	39.4 (1.2)	0.29
Infant sex (*n* male (%))	19 (54.3)	33 (51.6)	59 (56.2)	0.84
Infant birthweight (mean grams (SD))	3412.5 (537.8)	3451.7 (460.7)	3584.2 (403.4)	0.06
Infant birthweight (mean Z-score (SD))	− 0.02 (1.08)	− 0.02 (0.81)	0.28 (0.82)	0.04
Placental efficiency	− 0.09 (1.1)	− 0.10 (0.93)	0.10 (0.99)	0.37
Population	British Columbia, Canada	British Columbia, Canada	Queensland, Australia	
* Self-reported ethnicity (n (%))*				< 0.001
White	14 (40.0)	48 (75.0)	102 (97.1)	
Asian	12 (34.3)	6 (9.4)	1 (1.0)	
Black	0	1 (1.6)	1 (1.0)	
Other	8 (22.9)	1 (1.6)	0 (0.0)	
Missing	1 (2.9)	1 (1.6)	0 (0.0)	
Technical variables				
Processing time (median hours (SD))	2.9 (18.9)	28.7 (59.8)	Approximately 1 h	
Storage	− 20 °C	− 20 °C	Snap frozen liquid nitrogen	
DNA extraction method, location	Salt extraction, Vancouver	Salt extraction, Vancouver	Qiagen DNeasy blood and Tissue kit, Montreal, Canada	
Number of technical replicates	4	0	8	
* EPIC batch distribution (n (%))*				
Batch 1	0 (0.0)	64 (100.0)	94 (89.5)	
Batch 2	18 (51.4)	0 (0.0)	0 (0.0)	
Batch 3	16 (45.7)	0 (0.0)	11 (10.5)	

^*^p values are based on ANOVA for continuous variables and Chi-square tests for categorical variables

In addition to the analyses conducted on PlaNET epiphenotype variables, we note that although these three cohorts underwent similar sampling protocols and were processed for DNAme analysis at one center, there are some key between-cohort demographic differences (Table 1). First, maternal self-reported ethnicity/race (see Methods) differed between cohorts (*p* < 0.001), with almost all mothers from the QF2011 cohort identifying as white. Infant birth weight standard deviation (corrected for sex and gestational age) was also slightly higher in the QF2011 cohort (*p* = 0.04). Finally, sample processing and storage in the QF2011 cohort differed in subtle but potentially significant ways (see Methods). These key differences could contribute to cohort differences that were important to consider in data analysis.

### Main drivers of DNAme variation across cohorts

**Fig. 1:**
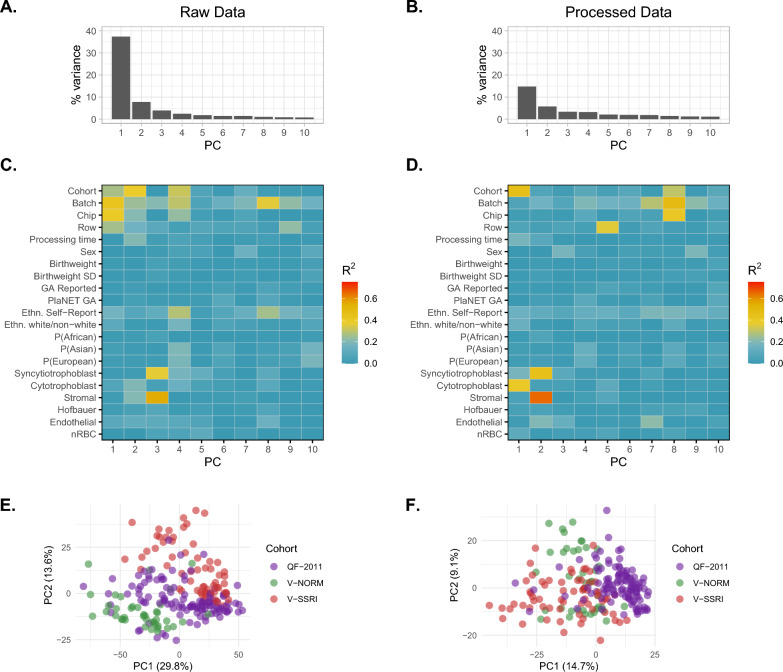
Principal component analysis of DNAme variation. Scree plots (**A**, **B**) show the proportion of variance explained by each principal component (PC), while heatmaps (**C**, **D**) show the *R*^2^ values of association from linear models run independently for each metadata variable (i.e., PC ~ Variable). **A** PC scree plot on raw data. **B** PC scree plot on processed, dasen + noob normalized data. **C** Raw data *R*^2^ heatmap showing strength of association between each PC from A and metadata variables. **D** Processed data *R*^2^ heatmap showing strength of association between each PC from B and metadata variables. For all plots, “SD” refers to standard deviation, “GA” refers to gestational age at birth, “Ethn” is used to denote ethnicity, P(African/Asian/European) represent the continuous probabilities from PlaNET ancestry prediction, and nRBC refers to nucleated red blood cells estimated by PlaNET. **E** Scatterplot of PC1 versus PC2 in the raw data, colored by cohort. **F** Scatterplot of PC1 versus PC2 in the processed data, colored by cohort

Before performing DNAme array data analysis, it is useful to assess the main drivers of DNAme variation in the raw and processed data. To that end, we used principal component analysis (PCA) in combination with linear models to assess the relationship between variation in the data (PCs) with major technical and biological variables (Fig. 1).

Our first main finding was that data processing (normalization and probe filtering) attenuated the association between DNAme variance and technical factors, and the reduced DNAme variance in the processed data was instead related to cohort and cell-type differences rather than technical factors. This was evidenced by the fact that in the raw data, the first two principal components (PC1 and PC2) accounted for nearly half (45.2%) of the DNAme variability across all samples, and were associated with cohort (*p* < 0.001 for Cohort = V-NORM, V-SSRI, or QF2011) and technical array variables (batch, chip, row, all *p* < 0.05) (Fig. 1). After data processing, the proportion of DNAme variation explained by PC1 and PC2 decreased to 15% and 6%, respectively, and PC1 was no longer strongly associated with batch, chip, or row effects. This was reassuring as we chose not to apply a batch correction step, which can introduce false signal, especially when there is an unequal distribution of variables of interest [36]. In the processed data PC1 remained significantly associated with cohort (*p* < 0.001, *R*^2^ > 0.25) and cytotrophoblast proportion (*p* value < 0.001, *R*^2^ > 0.25), while PC2 was also associated with cell type proportions. Array batch and PlaNET-derived ancestry were also weakly associated with PC1 and PC2 in the processed dataset (*p* values < 0.001, *R*^*2*^ < 0.25). The fact that PC1 was more strongly associated with cohort than any other variable suggests that there are unidentified technical and/or biological variables contributing to between-cohort variability. In summary, data normalization and probe filtering are essential for reducing DNAme variance associated with technical factors, and cohort, array batch, cell type proportion, self-reported ethnicity, ancestry, and sex are all important variables to consider in any downstream analyses of these data.

### Placental genetic ancestry epiphenotype accurately captures SNP genotype-estimated ancestry

**Fig. 2:**
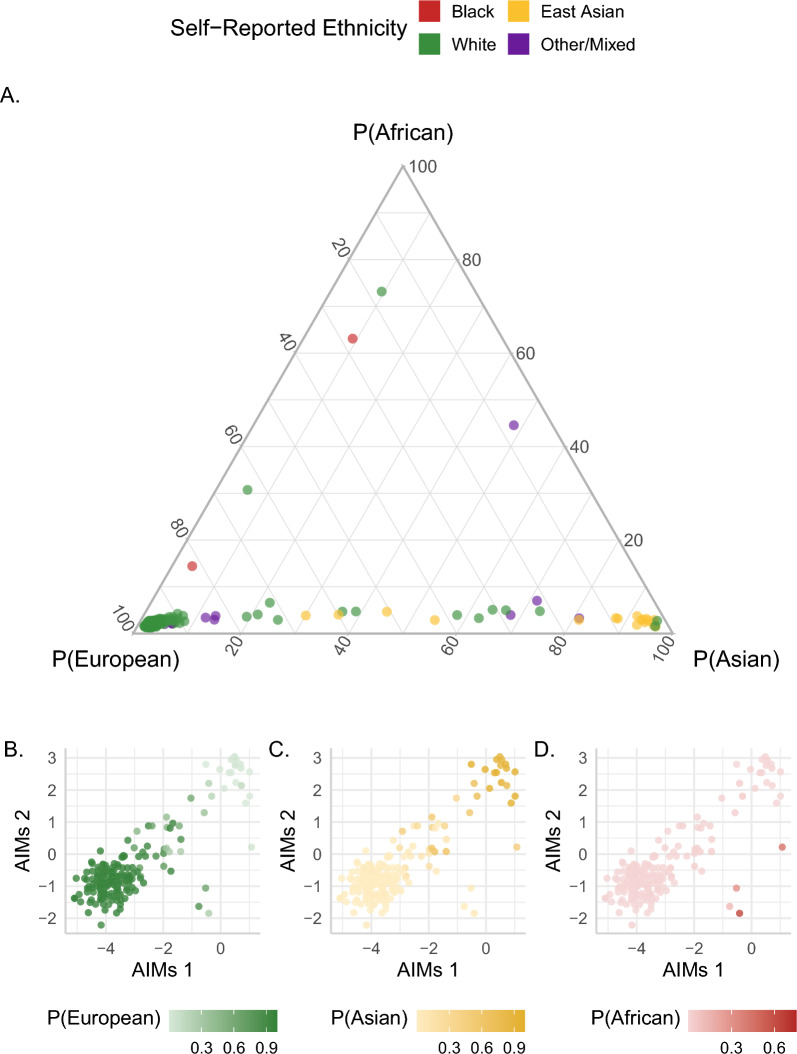
Relationship between PlaNET ancestry probabilities, self-reported maternal ethnicity, and placental Ancestry Informative Marker (AIMs) Coordinates. **A** Ternary plot of PlaNET ancestry probabilities (P(European), P(Asian) and P(African), colored by self-reported maternal ethnicity (Black, East Asian, white, other/mixed). Samples of unknown ethnicity were excluded. The three axis labels give 0 to 100 percent probabilities for samples belonging to each of the three ancestry groups. **B–D** Scatterplot of AIMs coordinates 1 (*x*-axis) and 2 (*y*-axis) colored by PlaNET ancestry probability score represented as a color gradient from 0 to 100 (green for P(European) (**B**), yellow for P(Asian) (**C**), and red for P(African) (**D**)). Samples of European ancestry tend to cluster in the lower left; Asian in the upper right, and African in the lower right by AIMs coordinates

DNAme variation is greatly influenced by genetic variation, which differs by ancestry of the individual. However, genetic ancestry data are often not measured, and while many pregnancy studies collect maternal self-reported ethnicity as an alternative measurement, this approach is inherently limited. First, ethnicity is a social concept that can be related to but is fundamentally different from genetic ancestry. Further, if only maternal ethnicity is collected, it ignores the other parent’s contribution to the placental genome and epigenome [37]. In addition, genetic ancestry is interesting to study in its own right, as it may independently drive DNAme variation and/or confound other associations. Previously, we created a tool to estimate genetic ancestry from the DNAme data directly (which is implemented in the PlaNET R package [29]), and here we compare this ancestry estimate to (i) maternal self-reported ethnicity (for details on ethnicity categories see Methods) and (ii) genetic ancestry assessed using Ancestry Informative Markers (AIMs), an independent set of SNPs that were genotyped for each placenta [38] (Fig. 2).

Most placentas (*n* = 172/204) had a high estimated probability (score > 75%) of European ancestry and for most of these (*n* = 151/172, 88%) the maternal self-reported ethnicity was “white”/European descent. All 17 placentas with a high probability (score > 75%) of East Asian ancestry had maternal self-reported “Asian” ethnicity, as did 4 additional samples (*n* = 21/204). No cases had a probability score > 75% of African ancestry, but of the 2/204 cases with a probability score > 50% of African ancestry, the maternal self-reported ethnicity was “Black” in one case. While there is a strong relationship between PlaNET ancestry estimates and maternal self-reported ethnicity, importantly, 24 placental samples (14% of the combined cohort) did not have values > 75% in any one ancestry dimension. This suggests a high degree of genetic diversity, which is important to consider in downstream analyses and cannot be captured by assigning samples to discrete ethnicities or ancestry groups. Looking at demographic and DNAme-derived variable relationships, we found that as expected, across all cohorts the various ethnicity and ancestry measures were associated (*R*^2^ 0.22–0.95) (Additional file 1: Fig. S2).

Although both PlaNET- and AIMs-inferred ancestry metrics yield continuous values in multiple dimensions of ancestry variation, the outputs of the two methods are not directly comparable. The two primary AIMs coordinates are sufficient to separate European, East Asian, and African ancestry samples, and were thus compared to the three PlaNET-derived ancestry probabilities (Fig. 2). In general, AIMS coordinates were found to correspond very well to PLANET ancestry probability scores, and most placentas that had values < 75% in the three PlaNET probabilities had AIMs scores in the first two coordinates in the mid-range of values. As few cases had high estimates for African ancestry by either method, we could not assess this ancestry dimension for interaction with other variables in subsequent analyses. As PlaNET ancestry probability is based on placental Illumina array data directly, it is a useful tool for considering genetic variation that influences DNAme variation, particularly when matched genotyping data are not available.

### The placental epigenetic clock can predict reported gestational age in term placentas

Gestational age at birth is often unavailable in public datasets, but this variable is important to account for in placental studies as DNAme changes dramatically with gestational age, even late in pregnancy [18]. Further, clinically reported gestational age, which is estimated by first trimester ultrasound (gold standard), later ultrasound, or based on self-report of last menstrual period (LMP), is associated with inherent variability [39, 40]. To address both of these problems, gestational age can be predicted from DNAme data itself, using several methods. Here, we applied the refined-robust placental clock (RRPC) as it was developed to estimate gestational age specifically for term placentas, which make up the vast majority of our cohorts [18].

**Fig. 3:**
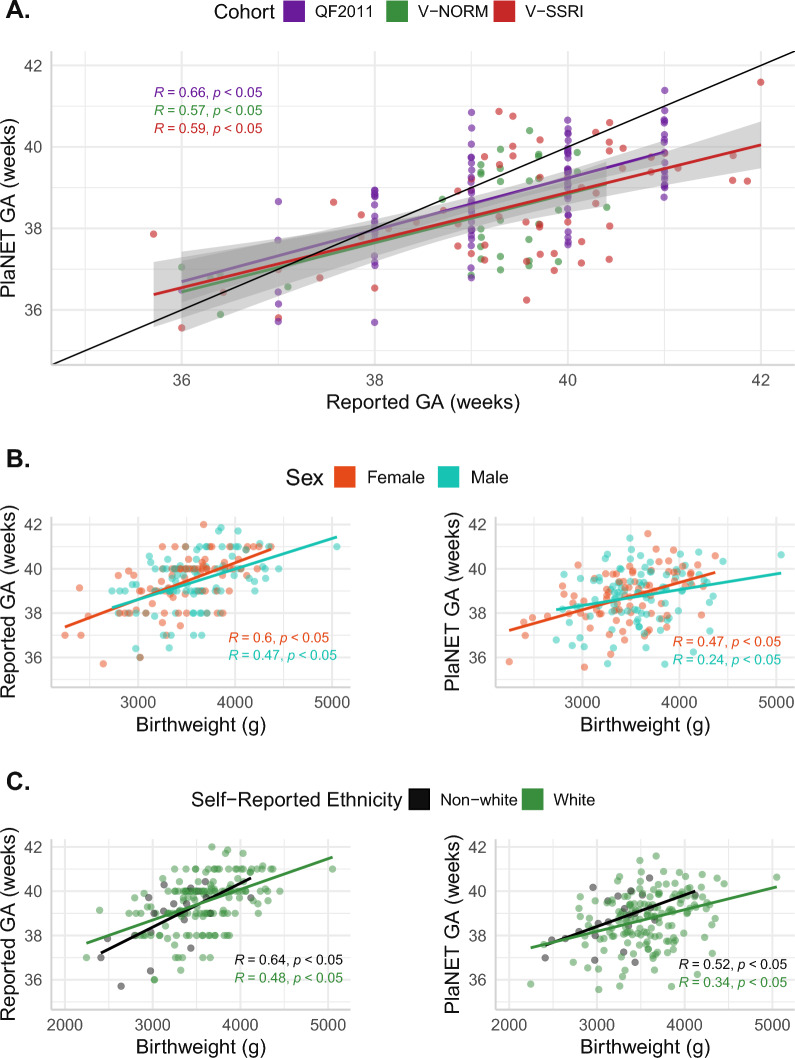
Relationships between PlaNET-estimated gestational age, clinically reported gestational age, sex, birth weight, and self-reported ethnicity. **A** PlaNET-estimated gestational age (GA) using the refined-robust placental clock, compared to clinically reported gestational age. Shading depicts the standard error of the estimate (95% confidence interval). **B** Reported and PlaNET-estimated GA versus birth weight, separated by sex. **C** Reported and PlaNET-estimated GA versus birth weight, separated by self-reported ethnicity

In each of the three cohorts, we observed a moderate correlation between reported and estimated gestational age (Pearson’s *R* = 0.54, 0.59, and 0.66 for V-NORM, V-SSRI, and QF2011, respectively). The median deviations between predicted and reported gestational age were < 1 week in all three cohorts (median deviations − 0.51, − 0.87, and − 0.57 weeks for V-NORM, V-SSRI, and QF2011, respectively) (Fig. 3). Considering the three cohorts together, the average median deviation between the RRPC and the reported gestational age was − 0.6 weeks, or − 4.3 days.

To further evaluate the gestational age epiphenotype, we compared both reported gestational age and RRPC-estimated gestational age to birth weight, as we expected both measurements of gestational age to correlate with infant size. Overall, clinically reported gestational age was more strongly correlated with birth weight than was the RRPC-estimated gestational age (Pearson’s *R* of 0.54 and 0.37, respectively) (Fig. 3). We found that these gestational age–birth weight relationships were not significantly different by sex or maternal ethnicity (white vs. non-white), although both measures of gestational age tended to be more strongly correlated with birth weight in placentas that were female or of non-white maternal ethnicity (Fig. 3). These results suggest that the RRPC-predicted gestational age is less accurate than clinically reported gestational age, at least in these cohorts, in which the range of gestational age at birth was small. However, these results also indicate that the RRPC is still quite accurate and could be used to predict gestational age when such data are missing, or when comparing gestational age across datasets, which may have different standards of gestational age estimation or reporting.

### Cell composition epiphenotype estimates can identify systematic differences between cohorts

The cell composition can vary between chorionic villi (bulk tissue) samples due to localized heterogeneity within the placenta, or due to systematic differences in sampling techniques between cohorts. As DNAme profiles vary markedly between cell types, they can be used to estimate the relative cell type proportions in whole chorionic villi samples (i.e., bulk tissue). Cell type proportions can then be compared between cohorts, datasets, or disease status groups to identify systematic between-group differences.

In assessing the inter-relationships between the different cell type proportions calculated with PlaNET, we found that cytotrophoblast proportion was inversely correlated (Pearson correlation) with syncytiotrophoblast proportion, and that there were no strong relationships between cytotrophoblasts or syncytiotrophoblasts and any other cell type proportions (Additional file 1: Fig. S2). The estimated proportions of Hofbauer cells and nRBCs, which are both typically very small, were also unrelated to other cell proportions.

**Fig. 4:**
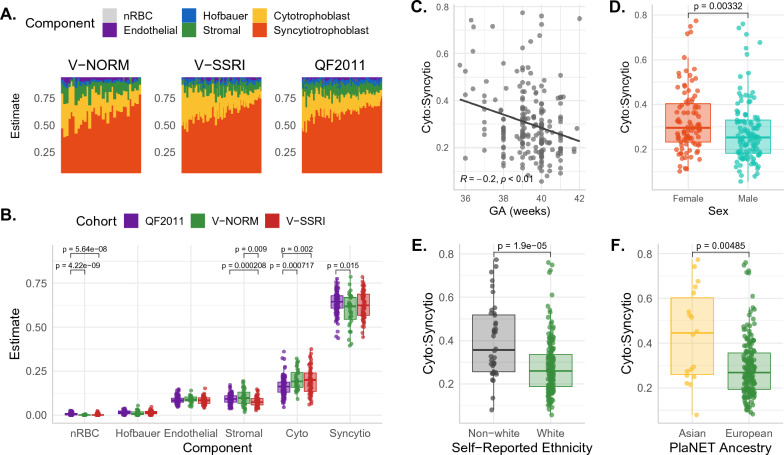
Association of cell type proportions with demographic variables. **A** PlaNET cell composition estimates across cohorts, shown as the estimated cell composition of each sample (column) by cohort. **B** The mean estimated proportion of each cell type separated by cohort. **C** Cytotrophoblast:syncytiotrophoblast ratio versus reported gestational age. **D** Cytotrophoblast:syncytiotrophoblast ratio by sex, **E** self-reported white ethnicity, and **F** PlaNET European or Asian ancestry probability > 0.75

The estimated distribution of major placental cell types was found to be similar across all three cohorts in our study (Fig. 4). We observed that the total proportion of trophoblasts (sum of syncytiotrophoblast and cytotrophoblast proportions) contributed to an average of 80.9% of each chorionic villus sample (SD = 3.6%; range 66.9–91.9%), while nucleated red blood cells (nRBCs) were present in only minor amounts (range 0.0–2.4%). The high trophoblast and low nRBC proportions confirm that, in these three cohorts, fetal blood contamination is negligible, and samples originate predominantly from the intermediate and terminal chorionic villi [15].

When comparing relative cell proportions across cohorts, some subtle but significant differences were noted. A slight decrease in stromal cells was observed in V-SSRI samples as compared to the other two cohorts (Fig. 4B). As the V-SSRI cohort was an outlier in that it contained multiple samples with very long processing times (> 100 h), we sought to evaluate whether processing time was associated with cell composition estimates (Additional file 1: Fig. S3). Increased processing time correlated with a reduction in stromal cell proportions, even when the SSRI dataset was removed to assess dataset-processing time confounds (*R* = − 0.37, *p* < 0.05). Beyond this small impact on stromal cells, however, processing time appeared to have little effect on placental cell composition estimates.

Samples from the QF2011 cohort displayed a slightly higher median estimate of syncytiotrophoblasts, and a lower median estimate of cytotrophoblasts, as compared to the other two cohorts (Fig. 4B), leading to a lower cytotrophoblast:syncytiotrophoblast ratio. We therefore sought to evaluate whether any demographic variables might also be associated with cytotrophoblast:syncytiotrophoblast ratio, and observed a lower ratio in association with increasing gestational age, male sex, white maternal ethnicity, and European PlaNET ancestry probability > 75% (Fig. 4C–F). To distinguish the impact of ancestry/ethnicity and fetal sex on cell types, as opposed to possible cohort effects (as the QF2011 cohort mainly included mothers that self-reported as white and placentas with European predicted ancestry) we investigated the associations between cytotrophoblast:syncytiotrophoblast ratio and maternal white ethnicity and fetal sex in the V-SSRI and V-NORM cohorts separately. Considering only these two cohorts, we found no association between sex and estimated cell proportions, however, lower cytotrophoblast:syncytiotrophoblast ratio remained associated with both maternal white ethnicity and with a high (> 75%) European ancestry probability (Additional file 1: Fig. S4). Because ethnicity has been reported to potentially associate with gestational age at birth [41], we hypothesized that the observed trophoblast ratio/ethnicity relationship may be arising from a confounding association between trophoblast ratio and gestational age. In these cohorts, we did observe a slightly lower gestational age at birth in cases of non-white versus white maternal ethnicity (*p* = 0.0012), and also observed a decrease in cytotrophoblast:syncytiotrophoblast ratio with increasing gestational age (*p* = 0.00052). However, when the linear model testing for association between white/non-white ethnicity and cytotrophoblast:syncytiotrophoblast ratio was adjusted for gestational age, trophoblast ratio remained associated with ethnicity (higher ratio in non-white ethnicity) (*p* = 3.18e−7).

### Further evaluation of relationships between epiphenotypes and biological and technical variables

Before performing statistical analysis, it is important to assess inter-relationships and possible collinearities between demographic and technical variables in a dataset, including any relevant epiphenotype variables, and any necessarily related demographic variables, such as birth weight and gestational age. As the datasets originally used to construct the PlaNET epiphenotyping tools may have inherent biases towards different technical or biological variables, investigating the relationships between these epiphenotypes and other dataset metrics in these three well-characterized cohorts could provide useful knowledge for future applications of these tools. Reassuringly, beyond the factors already discussed, we did not detect further associations between PlaNET epiphenotype variables and other biological or technical variables (Additional file 1: Fig. S2).

Of the remaining variables of interest, birth weight Z-score and placental efficiency (residual of fetal weight regressed on placental weight, sex, and gestational age) are both metrics of fetal growth during gestation, and are interesting to evaluate relative to the PlaNET tools for their relationships to both successful gestation and pathologic conditions such as preeclampsia or fetal growth restriction. Birthweight Z-score characterizes fetal growth by contextualizing infant birthweight relative to population-based reference groups of sex- and gestational-age-matched peers, while placental efficiency is a metric reflects the growth (mass) of a fetus relative to the growth (mass) of its own placenta. In principle, larger placentas can support larger infants, but the most “efficient” placentas are those that support adequate fetal growth with less relative placental mass. Birthweight Z-score and placental efficiency were significantly associated with each other, but were not strongly associated with other variables (Additional file 1: Fig. S2). It is worth noting, however, that birth weight Z-score and gestational age were both higher in placentas with high PlaNET European ancestry probability score (*p* < 0.001; *p* < 0.01, respectively), and in cases with maternal white ethnicity (*p* < 0.01; *p* < 0.001). We found that fetal:placental weight ratio was higher at lower gestational ages as reported in [42], and this ratio was thus also associated with altered cell type proportions (Additional file 1: Fig. S5). The residual of fetal weight regressed on placental weight, as an improved measure of placental efficiency, however, was not associated with either gestational age or cell type composition (Additional file 1: Fig. S5). Beyond the factors already discussed, we did not detect further associations between PlaNET epivariables and other biological or technical variables.

### Accounting for epiphenotypes in statistical analysis reduces test statistic inflation

As a final investigation into the utility of epiphenotype variables in analysis of placental DNAme data, we evaluated whether accounting for epiphenotypes during statistical analysis may help attenuate test statistic inflation (lambda) [43]. To that end, we produced a series of linear models testing for between-cohort DNAme differences across all filtered autosomal CpGs (*n* = 746,261), in all samples (*n* = 204). We also ran a series of equivalent models in only the V-NORM and V-SSRI samples (*n* = 99) as there was a systematic difference in the data collection method for gestational age between QF2011 (rounded to the nearest week) and the other two cohorts, see Methods.

The base model we assessed was adjusted for the additive covariates of sex and Sentrix Position (array row), and took the form: DNAme ~ Cohort + Sex + Sentrix Position + *ε*. We computed the lambda value across all p values obtained from this model, and compared this lambda to models iteratively adjusted for gestational age, RRPC gestational age epiphenotype, PlaNET ancestry, cell composition, and the combination of RRPC, ancestry, and cell composition. In the full dataset (*n* = 204), accounting for epiphenotypes of ancestry and cell type led to the largest reductions in lambda (approximately − 2 and − 8, respectively). In the series of models run on the V-SSRI and V-NORM samples only, accounting for reported gestational age had a similar magnitude of effect on lambda as did accounting for the RRPC gestational age epiphenotype (approximately − 0.5). See Additional file 1: Table S1, and Fig. S6.

The models run on the full dataset all had higher lambda values (median lambda = 19.26) than models run on the V-SSRI and V-NORM samples only (median lambda = 4.08). This example scenario does not reflect a complete analysis of the data, and is only meant to illustrate the impact of adjusting for epiphenotypes on epigenome-wide DNAme analysis. Typically, analysts would be investigating other outcomes with possibly different lambda values (beyond cohort effects), and given the lambda values obtained here, should also consider adjusting for unmeasured technical effects [43].

## Discussion

**Fig. 5:**
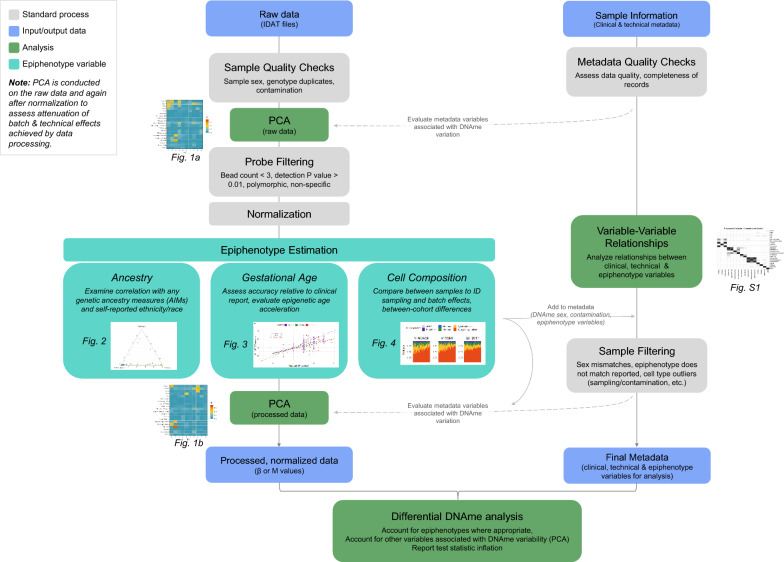
Suggested integration of placental epiphenotype variable estimation and analysis into DNAme processing and analysis pipelines. Each stage of the processing and analysis pipeline is depicted as a node in the flowchart, with node shading indicating extra information: standard processes are shaded grey, data are shaded blue, analysis stages are shaded green, and epiphenotype variable estimation steps are shaded teal. Where relevant, figures from this manuscript are referenced in this flowchart to indicate the stages at which they would be produced

In this analysis, we have demonstrated the applicability of placental epiphenotyping tools to independent data, and our findings indicate that these tools are appropriate for routine use in placental DNAme data processing and analysis pipelines in a variety of contexts. We have also reported how these epiphenotypes vary in association with extended sample characteristics such as gestational age, ethnicity, and processing time; our major findings in this regard are presented in Table 2. In summary, we find that placental epiphenotype variables first enable detailed technical assessment of data quality, for instance highlighting inter-cohort disparities, and sampling differences by comparing cell composition across samples. Next, epiphenotypes enable analysts to evaluate associated data accuracy by comparing each epiphenotype variable to analogous clinically reported data, such as comparing PlaNET ancestry to genetic ancestry (AIMs), or comparing epigenetic age to reported gestational age. Finally, we show that epiphenotype variables are useful covariates to include in downstream analysis, and they enable analysts to adjust for a portion of the unwanted variation that exists between datasets. We suggest the regular integration of epiphenotype variables into placental DNAme array data processing and analysis pipelines, and present our suggested framework for their inclusion in Fig. 5.

**Table 2:** Summary of findings in the evaluation of PlaNET package epiphenotyping tools for genetic ancestry, gestational age, and placental cell type composition

	PlaNET Epiphenotype variable		
	Ancestry	Gestational age (refined-robust clock, RRPC)	Cell composition
Validation	PlaNET ancestry is highly correlated with independent SNP genotype-derived ancestry-informative markers (AIMs)	The refined-robust placenta clock (RRPC) is less accurate than clinically reported gestational age for term placentas, but predicts gestational age on average within 4 days	Cell composition results as expected for term chorionic villi: e.g., 70–90% total trophoblast; < 2% nucleated red blood cells
Calculation considerations	PlaNET ancestry estimates are affected by normalization; data must be BMIQ + noob normalized prior to estimation [29]	Accuracy of placenta epigenetic clocks likely depends on data quality and uniformity of bulk tissue sampling	Recommended to estimate cell composition on BMIQ + noob normalized data [29]
Epiphenotype variation by:			
*Cohort of origin*	QF2011 has predominant European ancestry, while V-NORM and V-SSRI are more diverse	Similar across cohorts	Altered trophoblast ratio in QF2011 cohort
*Processing time*	No association	No association	Slight decrease in stromal cell composition at high processing times
*Sampling method*	Not evaluated	Not evaluated	Yes, previously reported [15], though not able to be evaluated in the present similarly-sampled cohorts
*Sex*	No association	No association	No association
*Ethnicity*	Correlated with maternal ethnicity; but ancestry includes paternal contribution and is continuous not categorical	No association	Possibly higher ratio of cytotrophoblast: syncytiotrophoblast in placentas with Asian ancestry
*Reported gestational age*	N/A	Accelerated epigenetic gestational age is reported in preeclampsia [18], though this work was conducted with an earlier placental epigenetic clock (not the RRPC)	Decrease in cytotrophoblast: syncytiotrophoblast ratio with increasing gestational age
*Cell composition*	No association	Decrease in the ratio of cytotrophoblast:syncytiotrophoblast with increasing gestational age, but RRPC gestational age is robust to cell composition	N/A

PlaNET can first be used to estimate ancestry along three continuous axes of variation. The main utility of these PlaNET ancestry probabilities is that they can account for ancestry-driven genetic variation that influences DNAme, particularly when genetic ancestry data are absent. Self-reported ethnicity is often collected and used as an estimate genetic ancestry, but even when both maternal and paternal ethnicity data are available for prenatal samples such as placenta, ethnicity is a categorical identity and a poor proxy for genetic ancestry, which is continuous in nature and more closely reflects genetic variation [44–46]. While human populations are much more diverse than can be captured on the three dimensions predicted by this tool (African, East-Asian, European) [44], it is a useful method for ancestry estimation when independent genetic data are unavailable. As DNAme data from more diverse populations becomes available, new tools can and should be created that improve upon the current ability to distinguish diverse genetic ancestries. It is also important to note that PlaNET or genetic ancestry estimates are not a substitute for self-reported ethnicity, which may be associated with important social determinants of health [46], including lifestyle and exposure factors interacting with the in utero environment. It is difficult to examine the effects of self-reported ethnicity independently from genetic ancestry, as the two are typically associated, though in this dataset, maternal ethnicity showed a slightly stronger association with PC1 (largest proportion of DNAme variation in the processed data) than did estimated ancestry. Both ancestry and ethnicity or race should be considered in DNAme analyses when applicable; many methylated sites are strongly associated with nearby genetic variants [47], and environmental effects (which may be captured by self-reported ethnicity) should be examined in the context of this underlying genetic variation.

The PlaNET placental gestational age clock (RRPC) was less strongly correlated with birth weight than was clinically reported gestational age, which implies reduced accuracy of the DNAme-derived estimate. Nonetheless, the RRPC gestational age on average deviated only by − 4.3 days from the reported gestational age, which is less than reported in the original publication of this tool (*r* = 0.26 with an absolute mean deviation of 7 days) [18]. The Lee et al. [40] study utilized publicly available placental DNAme datasets, and it is possible these were subject to variable quality of clinical records, which may explain the higher accuracy observed in our samples. In addition, gestational age measurements are approximations and are subject to variability even with the most modern measurement techniques, such as ultrasound, as a study of > 500,000 pregnant individuals in California reported that LMP-based gestational age had an absolute deviation > 14 days in 17.2% of cases compared to ultrasound-derived gestational age. Further, gestation length has been found to vary by self-reported race/ethnicity (based on studies from the United States and United Kingdom [48, 49]); we were not powered to see an effect of self-reported maternal ethnicity on placental epigenetic gestational age in these dataset, but it is possible that such an effect may be observable in more diverse cohorts.

While the present study focused on evaluating tools presented in the PlaNET R package, two other placental gestational age clocks, exist, developed by Mayne et al. [50] and Haftorn et al. [51]. The first was trained on publicly available placental DNAme datasets of diverse pathologies and has a median absolute deviation of (predicted–reported age) ± 1.47 weeks, while the Haftorn et al. clock was trained on placental samples from a well-characterized Finnish cohort, and had a mean absolute deviation of ± 3.6 days, similar to what we observed in the present study with the RRPC. Recently, we also observed much higher correlation (*R* = 0.94) between PlaNET-predicted gestational age (RPC) in a separate dataset with a broad range of gestational ages (25–38 weeks), also with median absolute error of 4 days [52]. Although reliable clinical data are best, application of the gestational age clocks provides a useful data check when epigenetic gestational age is compared to reported gestational age, and can aid in identifying potential metadata errors, or sample mix-ups. Additionally, epigenetic gestational age can provide an estimate of gestational age in cases where this information is not reported. For example, PlaNET-inferred gestational age was used to harmonize gestational age measurements across all samples in a study that combined multiple DNAme datasets where this information was missing [52]. In our analyses, we used the RRPC clock, which was developed specifically in term uncomplicated pregnancies. In contrast, the RPC clock may be more appropriate if samples include many preterm births, as this clock was trained on both control and pathological samples across gestation and was designed to be robust to pathology [18]. If researchers wish specifically to examine epigenetic gestational age acceleration, then it may be preferable to use the CPC clock, which was developed on uncomplicated “control” samples across a range of gestational ages. Using the CPC clock, we previously tested for an association between epigenetic age acceleration metrics and SSRI exposure or maternal mood (Hamilton Depression score) in the V-SSRI cohort, and found no association [53]. That said, the utility of these placental epigenetic clocks for studying epigenetic age acceleration is as yet largely unexplored and may have limited power due to the relatively short time frame of human gestation. Even in conditions such as preeclampsia which exhibit pathology associated with accelerated aging, the predicted gestational age is only slightly overestimated relative to reported gestational age [18, 50].

Regarding cell composition, the placenta is a heterogenous solid tissue with multiple cell types, derived from all three components of the blastocyst: (1) trophoblast from trophectoderm; (2) placental endothelial and endodermal cells from hypoblast [54, 55], and (3) Hofbauer cells from epiblast [15]. Each cell type in the placenta has a unique DNAme signature, which contributes to DNAme differences across samples of bulk chorionic villi [15]. Cell type composition differences are known to be a major source of variation in DNAme data in general, beyond just the placenta [56]. Although we could not validate our cell type proportion estimates (as the bulk of nuclei come from the multinucleated syncytiotrophoblast and accurate counts are not possible), the ratios observed here were consistent with our sampling technique, which aims to obtain consistent samples of free floating intermediate and terminal villi free of very large vessels and washed well of any contaminating blood [57, 58]. Using the PlaNET cell proportions has also been shown to be superior to reference-free approaches applied to the placenta [59]. Overall, based on cell proportions, placental sampling technique applied here appears to have been consistent between QF2011 (sampled in Brisbane, Australia) and the two Vancouver cohorts: V-NORM and V-SSRI. This between-cohort consistency in cell composition is reassuring, although in other studies we have observed large variations in cell composition between public datasets [15]. We thus suggest that when estimated placental cell composition indicates that total trophoblast proportions are significantly beyond the range of ~ 0.65–0.92, as seen here in our three representative cohorts, studies may benefit from sample removal to ensure homogeneous study groups, or the region of the placenta/method of sampling should be considered for possible interaction with results. Specifically, we anticipate that the proportions of total trophoblast, endothelial cells, and stromal cells in a sample may be related to sampling technique. For example, we have observed that trophoblast levels are lower if placental chorionic villi are sampled closer to the fetal-facing surface of the organ (immediately under the chorionic membrane), where larger vessels reside (stem villi) [15].

The cytotrophoblast:syncytiotrophoblast ratio was found to be strongly associated with gestational age over the last few weeks of gestation, which is consistent with the decrease of cytotrophoblast populations over time as these cells fuse to form the multinucleated syncytiotrophoblast, which in turn becomes increasingly abundant towards full term [15, 60]. The association between cytotrophoblast:syncytiotrophoblast ratio and maternal ethnicity/placental genetic ancestry (lower ratio in non-white and in low probability European placentas) can be partly explained by a reduced gestational age in these cases; however, given the association observed in our data between non-white ethnicity and reduced birth weight standard deviation (even when accounting for gestational age), socio-cultural influences may also be at play and should be explored in more depth in future studies. As placental pathology or environmental exposures may be associated with altered cell composition, which in turn contributes to changes in DNAme in bulk tissue, analysts should carefully consider how and when to correct for cell type composition in bulk tissues epigenetic analyses.

The inter-relationships between variables that affect placental DNAme are important to understand before undertaking further analysis. While we identified a small number of variables that differed between cohorts, including differences in ancestry composition and slight differences in trophoblast ratios, none of these factors individually explained as much variation in DNAme as the “cohort” variable itself did (significantly associated with PC1). This is an expected result, and cohort-level differences likely arise from the combination of many factors including different procedures used in sample processing, storage and DNA extraction, and differences in environmental exposure between samples comprising each cohort (e.g., diet, medication, environmental exposure, stress). This is particularly relevant as in this study, the QF2011 cohort was exposed to an acute environmental stressor (flood), which will be explored in a future study for its effect on DNAme. Although a subset of the V-SSRI cohort was exposed to SSRIs and gestational maternal depression, in a previous study we found no consistent signature of altered placental DNAme in association with these exposures, and thus this particular exposure variable is likely not a large driver of cohort-level differences [53].

Obstetrical outcomes can differ by the sex of the conceptus; for example, male (XY) placentas tend to be larger and more prone to proinflammatory response than female (XX) placentas [61]. Accordingly, we explored whether any epiphenotype variables were associated with sex, and found that overall sex was not strongly associated with ancestry, gestational age, or cell composition epiphenotype variables. Sex was also not associated with the first two principal components of DNAme variation in these three cohorts. Interestingly, a slightly higher cytotrophoblast:syncytiotrophoblast ratio was observed in female samples, but this effect was limited to the QF cohort (Additional file 1: Fig. S4). We did not observe sex differences in placental cell proportions in a previous study with a combined cohort size of *n* = 343 [16], implying that the observed higher female cytotrophoblast:syncytiotrophoblast ratio finding could either be due to statistical noise in these cohorts, or be related to unmeasured cohort-specific factors. Sex differences in DNAme [16, 62, 63] and gene expression profiles [64] have been observed at autosomal loci in the placenta, likely secondary to sex chromosome-related gene expression sex differences [16]. Additionally, a recent study indicated that placental DNAme patterns associated with gestational age may be driven by changes in cell composition across gestation, and suggested that these changes in cell composition across gestation may also differ between male and female placentas, although effect sizes were small [65]. Thus, if estimated placental cell composition and gestational age do vary by sex, this variation is likely of small effect size.

Cohort of origin, array batch, cell type proportions, self-reported ethnicity, genetic ancestry, and biological sex are important variables to consider in any analyses of Illumina DNAme data [66–68]. However, comprehensive evaluation of the impact of these factors on placental DNAme has not routinely been possible for various reasons, including lack of routine collection of genetic ancestry information (often only maternal ethnicity is collected), and the lack of cell type deconvolution algorithms for placenta, until very recently. Further, due to the unique DNAme profile of the placenta as compared to other tissues, epiphenotyping methods previously developed for application to blood and other somatic tissues are not applicable to studies of placental DNAme, as has been previously reported for the ancestry, age, and cell type deconvolution algorithms presented in PlaNET [15, 18, 33]. Estimating epiphenotype variables (gestational age, ancestry, cell proportions) from the placental DNAme data itself provides an independent check of the clinical data (thus flagging potential sample mix-ups or data errors) and can also provide a robust approach to compare metadata variables across different datasets with possibly different reporting standards (to identify systematic differences, e.g., in sampling approach). The epiphenotyping tools presented in PlaNET give researchers the potential to understand placental DNAme data more deeply, from both technical and biological perspectives. We note that the analyses presented in this work were conducted with data from the Infinium MethylationEPIC v1 array. Illumina has recently released the new MethylationEPIC v2 array, with expanded coverage of the genome (> 900,000 CpGs), and explicit exclusion of several poor-quality probes that are routinely removed from analysis of v1 data. While the PlaNET tools have not yet been validated with v2 placental DNAme data, the majority of CpGs that PlaNET utilizes for epiphenotype estimation are still present on the v2 array. For gestational age (all three clocks) and cell type (first and third trimester) estimation, 90% of the PlaNET probes are covered on the v2 array. The CpGs used by PlaNET for genetic ancestry estimation are only 63% covered by the v2 array, however, those CpGs included in the v2 array have significantly higher “importance” values for prediction classification than do the CpGs that are omitted from v2 (*p* < 0.05). While it still needs to be experimentally confirmed, this suggests that PlaNET ancestry estimation will still be a valid tool if applied to placental DNAme data collected with the v2 array.

We suggest integration of epiphenotype variables during both the processing and analysis of placental DNAme data; see Fig. 5. First, we recommend that analysts perform PCA on both the raw and processed data (after normalization & probe filtering), incorporating epivariables into the interpretation of the processed data PCA, to identify whether major axes of variation in a dataset are associated with factors such as cell composition. Beyond PCA, several other quality control steps, and data normalization, are important to perform to ensure the quality of the data (see Table 2), including analysis of the control probes included on the Illumina arrays, evaluation of the SNP probes, and fluorescence intensity checks [69, 70]. Data must be normalized by one of several different algorithms, the choice of which may depend on tissue or dataset [71]. Analysts may consider applying batch-correction tools to DNAme array data, though doing so can introduce unwanted noise and false signal in the data, particularly if sample sizes are small and/or all potential confounding variables are not very well balanced across rows, chips, and array batches [36, 72]. If any technical batch signals remain after data processing, as detected by examination of the epiphenotype variables or DNAme data directly, we suggest that these factors can be adjusted for in statistical modeling [73], or examined post hoc as possible confounders after data analysis.

As a proof-of-principle experiment, we demonstrated that accounting for epivariables in differential DNAme analysis of placental data can help attenuate test statistic inflation. This was demonstrated by running a series of linear models with “Cohort” as the primary variable of interest, and iteratively adjusting for the epiphenotype variables as additive covariates in these models. Small but progressive decreases in the lambda values, i.e., decreasing inflation of the p values, was observed with adjustment for epiphenotypes [43]. This reduction in lambda values was particularly apparent when cell type proportions were adjusted for, and was also observable most clearly in the reduced dataset models (V-NORM and V-SSRI samples only). Additionally, the difference in lambda values between the models run on the full dataset versus models run on the reduced dataset (QF2011 samples excluded) suggests that even small between-cohort differences can have a large impact on statistical analysis, and experiments should be designed with this in mind. Other approaches are also available to further attenuate technical effects during DNAme data analysis, and should be considered in placental research [43]. To encourage transparency, we also recommend analysts calculate and report the lambda inflation parameter in placental DNAme studies.

We note that the method by which biological and epiphenotype variables are accounted for in analyses following data processing should be carefully considered, given the associations observed here between cell composition, ethnicity, genetic ancestry, and gestational age/birth weight Z-score. Adjusting or “controlling” for these factors in statistical models can mask important relationships between these variables and the outcomes of interest. As factors such as cell composition, ethnicity, genetic ancestry, and gestational age/birth weight may all interact with fetal health in unique ways, they should be studied directly when possible. If sample size is sufficient, for example, data should be analyzed separately by maternal self-reported ethnicity groups, and by sex, since DNAme alterations associated with other variables of interest may differ within these groups. Though not explored here, it is also worth noting that epiphenotypes could be used for metadata harmonization across cohorts with different reporting standards (one could calculate the epigenetic gestational age for all samples and using these values in downstream analysis). We also note that these epiphenotype variables can be analyzed directly in relation to outcome variables of interest, such as disease status (e.g., Are cell type proportions altered in disease contexts? Does epigenetic age increase relative to reported gestational age in disease contexts?). We acknowledge that an important limitation of our study is the relatively small sample size limited to pregnancies at or near term. It will be interesting to explore these questions and further validation of the epivariables in larger and more diverse cohorts in the future.

Overall, we recommend the application of epiphenotyping approaches, followed by detailed exploration the interrelated nature of biological, technical, and epiphenotype variables in any dataset before beginning analysis, and further recommend that analysts exercise due caution in interpreting results.

## Materials and methods

### Cohorts

204 placentas from three cohorts were processed for DNAme arrays in Vancouver, Canada. The three cohorts consisted of: (i) V-NORM, a normative population of pregnancies recruited at BC Women’s Hospital (BCWH) in Vancouver, Canada (*n* = 35), as part of a study on Epigenetics in Pregnancy Complications (EPIC) [7, 74, 75]; (ii) V-SSRI, a population of pregnant individuals recruited in Vancouver, Canada, in the 20th week of gestation (*n* = 64), with/without clinical depression, and with/without the use of selective serotonin reuptake inhibitors (SSRIs) [53, 76]; and (iii) QF2011, a population of pregnancies exposed to a sudden-onset disaster during gestation due to catastrophic flooding in the Australian state of Queensland in early January 2011 (*n* = 105) [77]. Ethics approval for the V-NORM and V-SSRI cohorts, as well as overall approval for the present study was obtained by the University of British Columbia/Children’s and Women’s Health Centre of British Columbia Research Ethics Board (H04–70488, H12-00733, and H16-02280, respectively). The QF2011 study received ethics approval for the initial and follow-up protocols from the Mater Hospital Human Research Ethics Committee (1709 M, 1844 M). The QF2011 study also has ethics approval from the University of Queensland Human Research Ethics Committee (2013001236). For all cohorts, written informed consent was obtained from all participants, and all procedures complied with the ethical standards on human experimentation and with the Helsinki Declaration of 1975 (revised in 2008). A subset of V-NORM participants were recruited by the BC Children’s Hospital BioBank (BCCHB) (Vancouver, BC) an institutional biobank that collects samples and data from both children and women at BC Children’s and Women’s Hospitals and Health Centres for future, ethically approved research.

The V-SSRI and QF2011 were prospectively recruited cohorts, and, except for four individuals giving birth between 35.7 and 37.0 weeks, all births occurred at term. Cases for V-NORM were thus retrospectively selected for having similar gestational ages at birth (i.e., ≥ 36 weeks) to match the other two cohorts, with four samples included between 36 and 37 weeks gestational age. Of note, gestational age was only available to the nearest week for the QF2011 cohort, with three missing values in the 105 placentas that were imputed to the median of all other measurements (39 weeks). Exclusion criteria applied to all cohorts included pregnancies with multiple fetuses and chromosome abnormalities. Additionally, V-SSRI excluded mothers with bipolar illnesses, hypertension, current substance abuse, any diabetes, or infants with congenital brain malformations or fetal growth. V-NORM excluded any pregnancies affected by preeclampsia, while QF2011 was not subject to any additional specific exclusions. The respective exclusion criteria were applied to all cohorts to enable examination of key variables of interest without the presence of large confounding factors (such as chromosome abnormalities or preeclampsia being associated with DNAme alterations, or bipolar illness possibly confounding depression analyses in the V-SSRI study).

Self-reported ethnicity and/or race are increasingly recognized as important variables to consider in health research, but there have not been consensus definitions of race or ethnicity [78–80]. Further, socially meaningful groupings may differ across countries and cultures, or even change for an individual over time [78]. To harmonize these self-reported variables across cohorts, as well as to create groups with sufficient sample size for analysis, we have chosen to group samples by maternal self-declared race/ethnicity as follows: (i) “white” if reported as white, Caucasian, European, or from any European country; (ii)“Asian” if reported as Asian, Chinese, Japanese, Korean, Filipino, Vietnamese or Thai; (iii)“Black” if reported as Black or African; (iv) and “Other” if reported as Pacific Islander, South Asian, South American, Middle Eastern, Latin American, any specific country within those areas, or mixed ethnicity. We acknowledge, however, that these are imperfect descriptors, and that these groupings may not accurately reflect the intended response of the participants.

Infant birth weight is presented as standard deviation Z-scores from the mean sex- and gestational age-specific birth weights, based on Canadian birth charts [81]. Placental efficiency was calculated as the residual of birth weight regressed on placental weight, adjusted for gestational age and sex [42]. This residual is independent of gestational age, whereas infant birth weight to placental weight ratio is positively correlated with gestational age [42]. Untrimmed placental weight (placental weight including the reflected amniotic and chorionic membranes), rather than trimmed weight, was used for placental efficiency calculations as it was available in a greater number of cases, and the trimmed and untrimmed values were highly correlated in cases for which both measurements were available (*n* = 75, Spearman’s Rho = 0.97, *p* < 2.2e−16). Between-cohort differences were evaluated by ANOVA for continuous variables and Chi-square tests for categorical variables.

### Placental sampling

Placental sampling after delivery followed two similar but distinct sampling processes. First, the V-NORM and V-SSRI cohorts were sampled by a single lab in Vancouver, Canada, using a standardized sampling protocol [75]. Briefly, 1.5–2 cm^3^ samples of chorionic villi were taken from each of four distinct cotyledons (sites) from below the surface of the fetal-facing side of the placental disc at a depth that targeted intermediate and tertiary villi and well-washed of blood. Any potential contaminating maternal tissue (i.e., decidua or maternal infarcts) was carefully avoided. Placental processing time (number of hours from placenta delivery until sampling) ranged from 0.5 to 288 h (with 5 samples missing data). The samples were washed thoroughly to remove blood, and any thick vessels were removed. Samples were frozen at − 20 °C until use. DNA was then extracted from all four cotyledon samples using a salting-out DNA extraction procedure [82], and extracted DNA from the four sites was pooled in equimolar proportions to provide a representative sample of each placenta. The second sampling process involved the QF2011 placentas, which were processed in Brisbane, Australia, within 60 min of delivery, and eight sites (1 cm^3^ each) representing different cotyledons were sampled across the fetal-facing side of each placenta. These samples were snap-frozen in liquid nitrogen and subsequently shipped to Montreal, Canada. Pools of five samples were ground over dry ice, and DNA was extracted using the DNeasy Blood & Tissue Kit (Qiagen, Valencia, CA, USA) in Montreal before being shipped to Vancouver on dry ice for DNAme processing.

### DNAme arrays and data quality checks

DNA samples from all three cohorts were run on Illumina Infinium MethylationEPIC v1 arrays in Vancouver, BC, Canada. Processing included DNA purification after extraction using the DNeasy Blood & Tissue Kit (Qiagen, Valencia, CA, USA), bisulfite conversion using the EZ DNAme Kit (Zymo Research, Orange, CA, USA), and hybridization to and processing of the Illumina Infinium MethylationEPIC BeadChip arrays according to the manufacturer’s protocol (Illumina, San Diego, CA, USA). Samples from the three cohorts were distributed and run in 3 array batches across 44 eight-sample chips as illustrated in Additional file 1: Fig. S1. Samples were carefully distributed across array chips (1–44) and rows (1–8) with respect to the following variables, to minimize potential batch effects: exposure groups (SSRI exposed/non-exposed and QF2011 objective flood stress high/low) and infant sex (all cohorts). All samples from V-SSRI and the majority of samples from QF2011 were run together in EPIC array Batch 1. A small number *(n* = 11) of QF2011 samples were received in Vancouver at a later date and were included in Batch 2. All samples from V-NORM were run in EPIC array batches 2 and 3, along with placental samples that were part of other, related projects.

DNAme data from raw IDAT files were read into R v4.2.2 [83] and annotated with the Illumina Infinium MethylationEPIC v1.0 B4 Manifest. Several data quality control checks were undertaken using the R packages minfi [84, 85], wateRmelon [86, 87]*,* and ewastools [69]. First, each sample was assessed at 17 Illumina control probes to evaluate bisulfite conversion efficiency and array run quality; all samples passed the manufacturer-recommended thresholds at the control probes. Next, average total (methylated + unmethylated) fluorescence intensity was assessed between samples, and between array batches. All samples had similar total fluorescence, though samples run on the EPIC array in Batch 3 had slightly higher average intensities than those in Batch 1 and 2. Sample sex was assessed with the ewastools package [69], using the mean total fluorescence intensity (methylated + unmethylated) of the *X* and *Y* chromosome probes, normalized to the per-sample mean autosomal total fluorescence intensity, and was confirmed to match the clinically reported sex of the infant in all cases. Sample genetic identity was assessed using the 59 SNP (‘rs’) probes on the EPIC array with the “call_genotypes” and “enumerate_sample_donors” functions (*ewastools*) [69]. Finally, DNAme beta value density plots of all samples were visually assessed to determine overall similarity of the beta value distributions between samples, with no outliers identified.

### Epiphenotype estimation

The PlaNET *R* package [29] was used to determine DNAme-based estimates of genetic ancestry, placental cell type composition, and gestational age at birth. These metrics were calculated based on BMIQ-noob normalized data before probe filtering, as recommended in the PlaNET package documentation [29]. *PlaNET-derived genetic ancestry* can be described as a continuous variable on three axes of variation that sum to one, representing contributions of African, East-Asian, and European ancestry [33]. *PlaNET-derived cell composition* was calculated using the robust partial corrections method, which yields six compositional estimates of the major placental cell types (endothelial cells, stromal cells, Hofbauer cells, nucleated red blood cells, cytotrophoblasts, and syncytiotrophoblasts) [15]. To avoid confusion, we use the term “Cytotrophoblast” for the cell type PlaNET reports as “Trophoblasts”, as these were single-nuclear trophoblasts derived from chorionic villi, and represent stem and columnar cytotrophoblast, but would not be expected to have significant contribution from extra-villous trophoblast or syncytiotrophoblast [15]. *PlaNET-derived gestational age* can be calculated using 3 different built-in tools: the robust placental clock (RPC), the control placental clock (CPC), and the refined-robust placental clock (RRPC). The RRPC is most appropriate to the present dataset as it was developed using exclusively samples > 36 weeks of gestational age (including pathological samples) to improve prediction over the narrow age range at term [18].

### Data processing

After estimation of epiphenotype variables, raw data were normalized for analysis, using noob and dasen combined normalization methods [86, 88]. Several normalization algorithms were considered for application to this dataset (functional, BMIQ, SWAN, and quantile, all evaluated with and without noob where possible [70, 84, 89–91]). We selected between these algorithms by assessing the extent to which each normalization procedure adjusted the dynamic range of Type II probes to more closely resemble the distribution of Type I probes. Quantitatively, we compared the shape of the Type I and Type II probe beta value distributions by computing the difference between the maxima and minima before versus after normalization. Dasen + noob was found to outperform other normalization approaches in this dataset with respect to minimizing the difference between the distributions of Type I and Type II probes and increasing the correlation of technical replicate pairs after normalization.. Subsequently, poor-quality probes (detection *p* value > 0.01 or bead count < 3 or missing values in > 5% of samples) were removed from the dataset (*n* = 4783), as were cross-hybridizing probes and probes overlapping single-nucleotide polymorphisms (MASK_general column of [92], *n* = 99,360). Technical replicates of 12 genetically distinct samples (11 replicate pairs and one sample run in quintuplicate) were used to assess data processing by calculating the correlation between all DNAme beta values of replicate sample pairs in the raw and processed datasets. The highest quality replicate from each pair was retained for the rest of the analysis, and all others removed (*n* = 15 replicate samples removed). One additional non-replicate sample was removed for failing probe quality checks (> 1% of array probes failed detection P/bead count). After data processing and quality control, a total of 746,608 probes in 204 samples remained for analysis. Of note, we elected not to apply *ComBat* [93], a commonly used batch-correction tool, as our samples were well-distributed across the technical (array) batches with respect to biological variables (cohort, infant sex, and exposure groups: SSRI exposure and objective flood stress), and *ComBat* can lead to false positive discoveries [36, 72].

### Principal component analysis

*Principal component analysis (PCA)* was used to assess the primary drivers of DNAme variance in the data using the *R* package irlba [94]. Linear models were run to assess covariance between each principal component and technical and biological variables (PC ~ dependent variable) using the plomics package [95], and visualized in a heatmap method similar to [96].

### AIMs data processing

*Ancestry informative markers (AIMS)* were used as an independent assessment of genetic ancestry. Genotypes at 57 single-nucleotide polymorphisms informative to assess African, East Asian, and European ancestry [38, 97] were obtained using the Sequenom iPlexGold assay for 192/204 samples, and analyzed as previously described [38]. Briefly, for each sample individually, AIMS data were combined with external data from 2418 individuals from the 1000 Genomes Project (1 KGP), serving as ancestry reference populations. Multidimensional scaling (MDS) was then run on the Euclidean distance matrix based on genotype of these samples (coded numerically by the B allele frequency as 0, 1, or 2). The top two MDS coordinates were used to describe ancestry for each sample across a continuum relative to 1 KGP samples of East-Asian, African, and European ancestry, and are denoted throughout the article as AIMs coordinates.

### Evaluation of test-statistic inflation in linear models accounting for epivariables

DNAme data at all filtered autosomal CpGs (*n* = 746,261) were converted to M values, and subjected to linear modeling, testing for between-cohort DNAme differences, with sex and Sentrix Position (array row) included as additive covariates. Subsequent linear models were run that included additional additive covariates beyond sex and Sentrix Position: reported gestational age, RRPC gestational age epiphenotype, PlaNET ancestry estimates as continuous values, and PlaNET cell composition estimates as continuous values. The two compositional epiphenotype variables (ancestry and cell composition) were adjusted for using a “leave one out” structure, where all but one continuous variable from each epivariable set were included in the linear models (for example, cell type estimates were adjusted for by including additive covariates of all cell types except syncytiotrophoblast (cytotrophoblast, stromal, Hofbauer, endothelial, nucleated red blood cells), as recommended in the package vignette [29]). Linear models were run using the limma *R* package [98], and lambda values were calculated from the full set of “Cohort” term p values for each linear model using the QCEWAS *R* package [99].

### Supplementary Information

**Additional file 1: Fig. S1. Sample map for EPIC array processing.** Depiction of sample distribution across Illumina Infinium MethylationEPIC array chips, colored by randomization variables (sex, SSRI exposure status, COSMOSS stress score, replicate status, trimester, cell type). Chips are grouped by batch. **Fig. S2.** Heatmap of the strength of association between pairs of covariates. R^2^ values of linear models run on Covariate ~ Covariate demographic variables. “Ethn” denotes ethnicity, “P(African/Asian/European)” refer to the continuous PlaNET ancestry probabilities, “SD” refers to standard deviation, “wt” refers to weight, “GA” refers to gestational age at birth, “Cyto” refers to cytotrophoblast, and “nRBC” refers to nucleated red blood cells. **Fig. S3. Relationship between processing time and cell type proportions**. (A) Placental processing time in hours after delivery (Proc time) is plotted along the Y axis, with cohort plotted along the X axis. (b) Estimates of cell type proportions (Y axis) were plotted against placenta processing time (hours) from all cohorts. Significant Pearson correlations (Estimate ~ Cell Type) are indicated with p < 0.05 in the figure legend. (C) Samples from the V-SSRI cohort were excluded, to evaluate the impact of processing time on cell type proportions independent of the few samples in V-SSRI with unusually long processing times. Significant Pearson correlations are indicated with p < 0.05 if the figure legend. **Fig. S4. Relationship between cell type proportions and sex, self-reported maternal ethnicity, and PlaNET ancestry.** (A, C, E) All Cohorts, (B,D,F) Vancouver-collected cohorts only, QF2011 cohort excluded. Significance of comparisons are indicated when p < 0.05. **Fig. S5. Relationship between cell type proportions and placental to fetal weight ratio and residual.** (A) Fetal to placental weight ratio association with cell type proportions. Significant correlations are indicated with p < 0.05 in the legend. (B) Residual of fetal weight regressed on placental weight showed no significant association with any cell type proportion. **Fig. S6. Distribution of all nominal p values for linear models run with adjustment for epiphenotype variables.** “Base” refers to the base linear model of DNAme ~ Cohort + Sentrix Position + Sex + ε. Additional models refer to the base model plus the specified additive covariate. For example, GA (gestational age) refers to DNAme ~ Cohort + Sentrix Position + Sex + GA + ε. P values investigated are those associated with the term “Cohort”. RRPC indicates robust refined placental clock, Ancestry refers to adjustment for PlaNET ancestry continuous values, Cells refers to adjustment for continuous PlaNET cell composition estimates. Listing > 1 variable indicates additive adjustment for all indicated variables (such as adjustment for both ancestry and cell composition as indicated by the notation Ancestry_Cells). A horizontal dashed line indicates p = 0.05. The p values shown in this plot arise from linear models run on V-SSRI and V-NORM (n = 99) at all filtered autosomal CpGs. **Table S1. Lambda values from linear models for differential DNAme by Cohort.** Lambda was calculated in each case from all nominal p values associated with the Cohort term in each model. GA refers to gestational age, RRPC refers to the robust refined placental clock gestational age, Ancestry and Cell Types refer to the PlaNET epiphenotype variables for ancestry and cell composition, included as continuous additive covariates.

## Data Availability

The data supporting the conclusions of this article are available in the Gene Expression Omnibus repository under accession GSE232778 in both raw and processed form. The raw data have been uploaded without inclusion of the EPIC ‘rs’ genotyping probes to protect participants’ genetic privacy, though this information is available upon reasonable request to the authors.
