# Supplementary material for: The application of epiphenotyping approaches to DNA methylation array studies of the human placenta
Source: Epigenetics Chromatin. 2023 Oct 4;16:37. doi: 10.1186/s13072-023-00507-5 (PMC10548571; doi:10.1186/s13072-023-00507-5)
Supplement: Supplementary file 1 — Additional file 1: Fig. S1. Sample map for EPIC array processing. Depiction of sample distribution across Illumina Infinium MethylationEPIC array chips, colored by randomization variables (sex, SSRI exposure status, COSMOSS stress score, replicate status, trimester, cell type). Chips are grouped by batch. Fig. S2. Heatmap of the strength of association between pairs of covariates. R2 values of linear models run on Covariate ~ Covariate demographic variables. “Ethn” denotes ethnicity, “P(African/Asian/European)” refer to the continuous PlaNET ancestry probabilities, “SD” refers to standard deviation, “wt” refers to weight, “GA” refers to gestational age at birth, “Cyto” refers to cytotrophoblast, and “nRBC” refers to nucleated red blood cells. Fig. S3. Relationship between processing time and cell type proportions. (A) Placental processing time in hours after delivery (Proc time) is plotted along the Y axis, with cohort plotted along the X axis. (b) Estimates of cell type proportions (Y axis) were plotted against placenta processing time (hours) from all cohorts. Significant Pearson correlations (Estimate ~ Cell Type) are indicated with p < 0.05 in the figure legend. (C) Samples from the V-SSRI cohort were excluded, to evaluate the impact of processing time on cell type proportions independent of the few samples in V-SSRI with unusually long processing times. Significant Pearson correlations are indicated with p < 0.05 if the figure legend. Fig. S4. Relationship between cell type proportions and sex, self-reported maternal ethnicity, and PlaNET ancestry. (A, C, E) All Cohorts, (B,D,F) Vancouver-collected cohorts only, QF2011 cohort excluded. Significance of comparisons are indicated when p < 0.05. Fig. S5. Relationship between cell type proportions and placental to fetal weight ratio and residual. (A) Fetal to placental weight ratio association with cell type proportions. Significant correlations are indicated with p < 0.05 in the legend. (B) Residual of fetal weight [file 13072_2023_507_MOESM1_ESM.docx]

## Additional file Figures

**Additional file 1: Fig. S1. Sample map for EPIC array processing.** Depiction of sample distribution across Illumina Infinium MethylationEPIC array chips, colored by randomization variables (sex, SSRI exposure status, COSMOSS stress score, replicate status, trimester, cell type). Chips are grouped by batch.


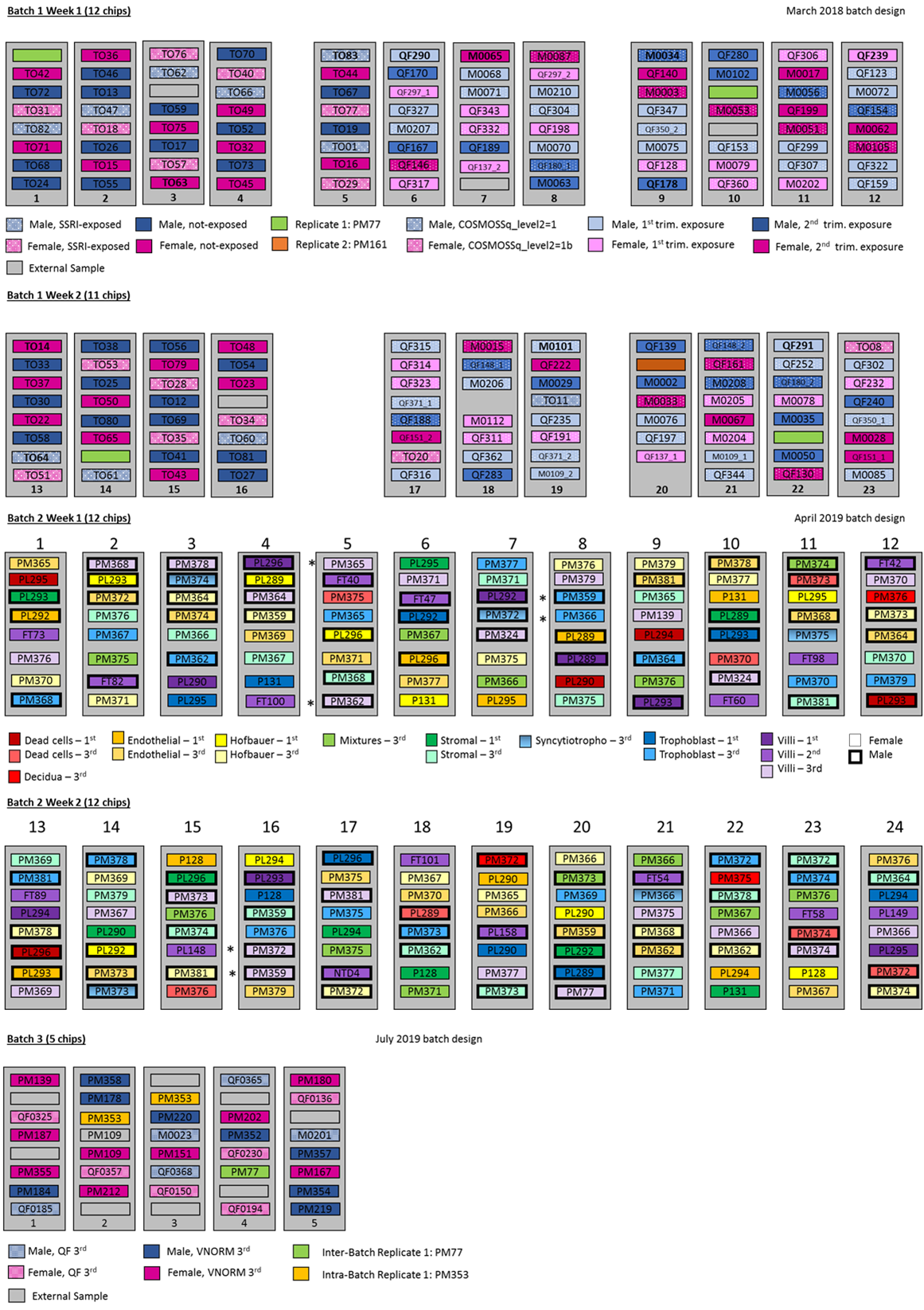


Additional file 1: Fig. S2. Heatmap of the strength of association between pairs of covariates. R^2^ values of linear models run on Covariate~Covariate demographic variables. “Ethn” denotes ethnicity, “P(African/Asian/European)” refer to the continuous PlaNET ancestry probabilities, “SD” refers to standard deviation, “wt” refers to weight, “GA” refers to gestational age at birth, “Cyto” refers to cytotrophoblast, and “nRBC” refers to nucleated red blood cells.

**
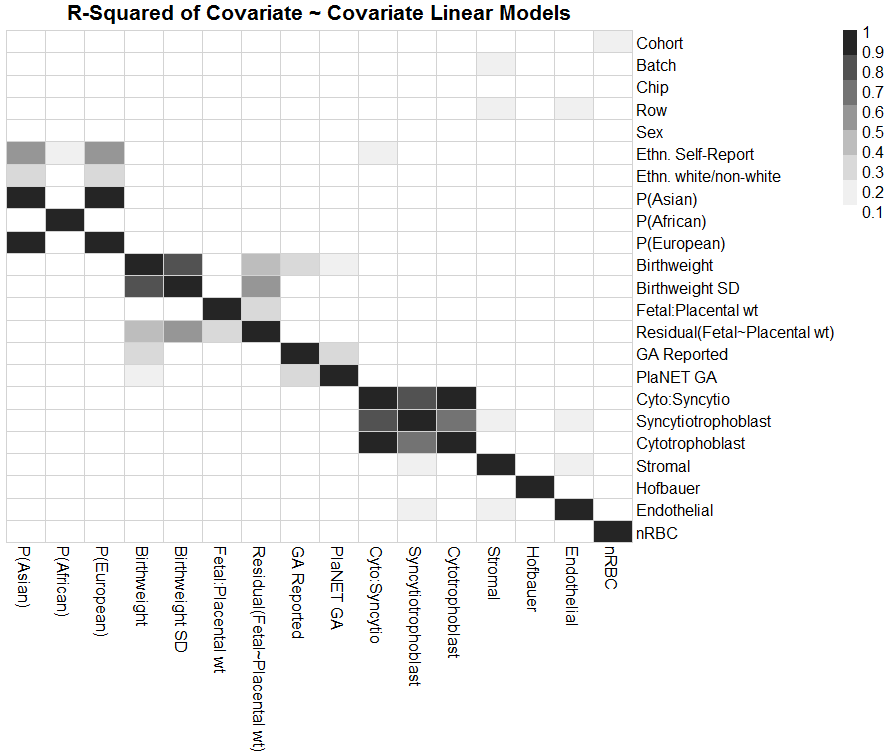
**

**Additional file 1: Fig. S3. Relationship between processing time and cell type proportions**. (A) Placental processing time in hours after delivery (Proc time) is plotted along the Y axis, with cohort plotted along the X axis. (b) Estimates of cell type proportions (Y axis) were plotted against placenta processing time (hours) from all cohorts. Significant Pearson correlations (Estimate ~ Cell Type) are indicated with p < 0.05 in the figure legend. (C) Samples from the V-SSRI cohort were excluded, to evaluate the impact of processing time on cell type proportions independent of the few samples in V-SSRI with unusually long processing times. Significant Pearson correlations are indicated with p < 0.05 if the figure legend.

**
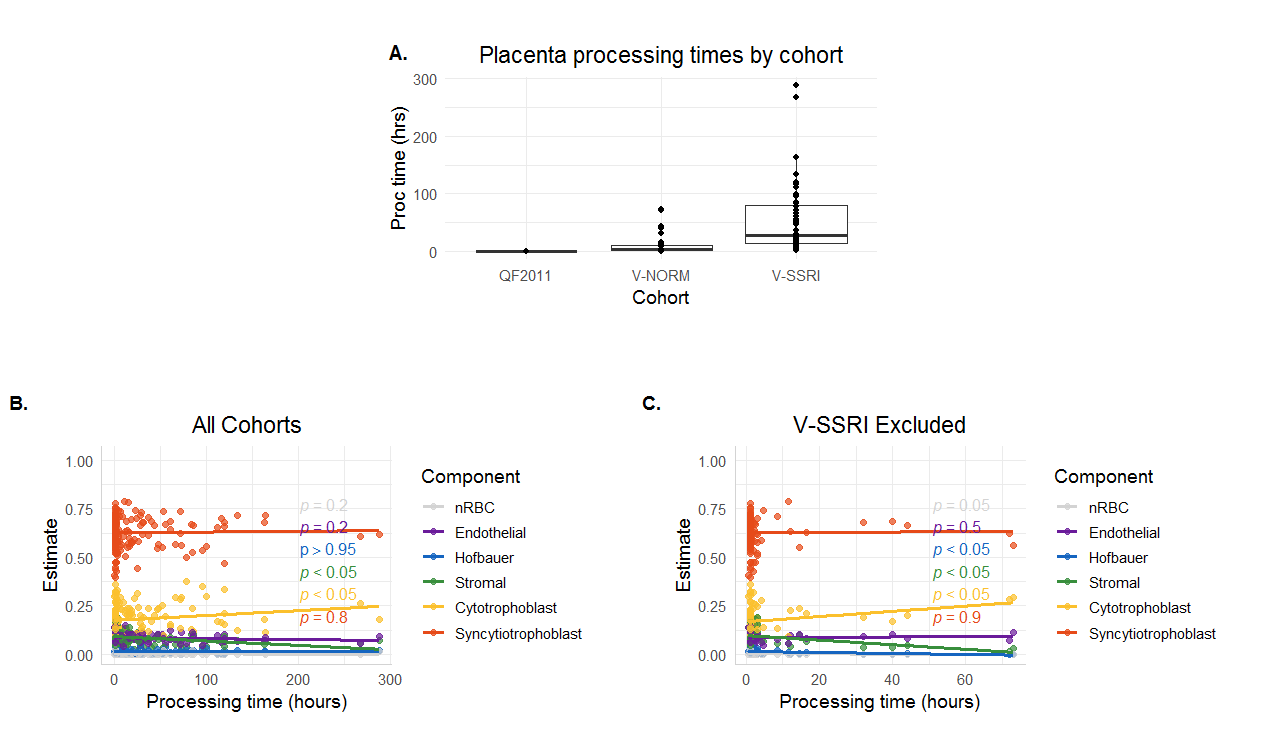
**

Additional file 1: Fig. S4. Relationship between cell type proportions and sex, self-reported maternal ethnicity, and PlaNET ancestry. (A, C, E) All Cohorts, (B,D,F) Vancouver-collected cohorts only, QF2011 cohort excluded. Significance of comparisons are indicated when p < 0.05.

Additional file 1: Fig. S5. Relationship between cell type proportions and placental to fetal weight ratio and residual. (A) Fetal to placental weight ratio association with cell type proportions. Significant correlations are indicated with p<0.05 in the legend. (B) Residual of fetal weight regressed on placental weight showed no significant association with any cell type proportion.


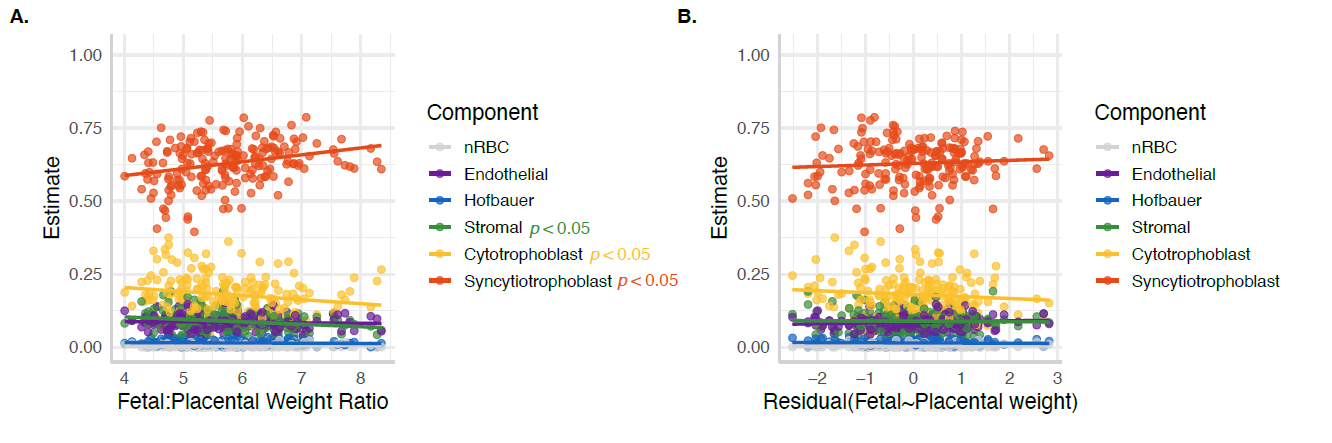


**Additional file 1: Fig. S6.** **Distribution of all nominal p values for linear models run with adjustment for epiphenotype variables.** “Base” refers to the base linear model of DNAme ~ Cohort + Sentrix Position + Sex + ε. Additional models refer to the base model plus the specified additive covariate. For example, GA (gestational age) refers to DNAme ~ Cohort + Sentrix Position + Sex + GA + ε. P values investigated are those associated with the term “Cohort”. RRPC indicates robust refined placental clock, Ancestry refers to adjustment for PlaNET ancestry continuous values, Cells refers to adjustment for continuous PlaNET cell composition estimates. Listing > 1 variable indicates additive adjustment for all indicated variables (such as adjustment for both ancestry and cell composition as indicated by the notation Ancestry_Cells). A horizontal dashed line indicates p = 0.05. The p values shown in this plot arise from linear models run on V-SSRI and V-NORM (n=99) at all filtered autosomal CpGs.


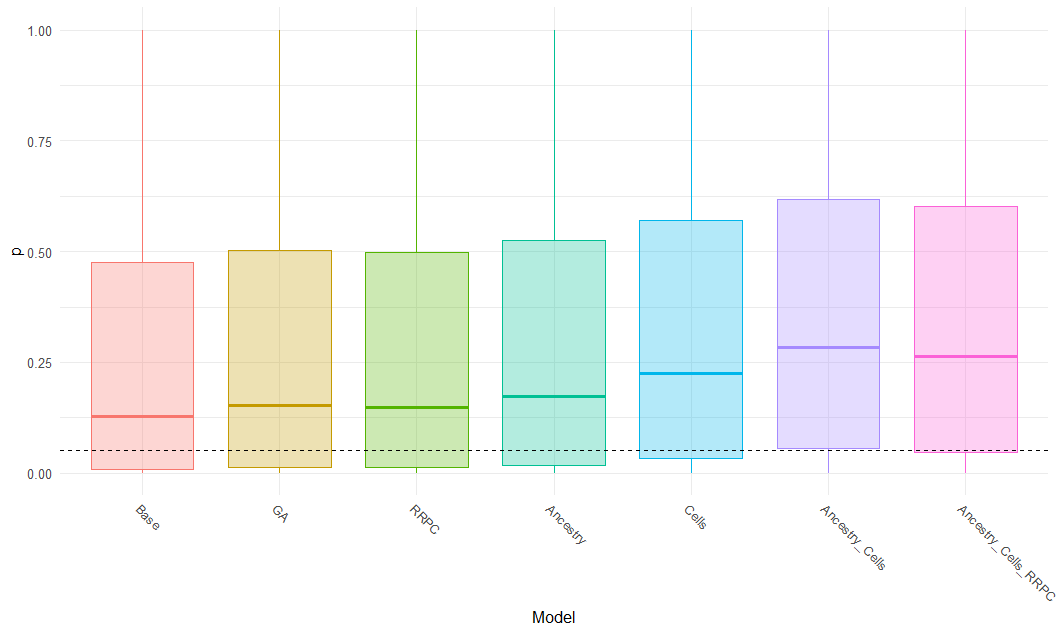


## Additional file Tables

**Additional file 1: Table S1. Lambda values from linear models for differential DNAme by Cohort.** Lambda was calculated in each case from all nominal p values associated with the Cohort term in each model. GA refers to gestational age, RRPC refers to the robust refined placental clock gestational age, Ancestry and Cell Types refer to the PlaNET epiphenotype variables for ancestry and cell composition, included as continuous additive covariates.

| Description | Model | Inflation of p values (lambda)  *V-NORM + V-SSRI + QF2011* | Inflation of p values (lambda)  *V-NORM + V-SSRI* |
| --- | --- | --- | --- |
| Base model | DNAme ~ Cohort + Sex + Row + ε | 21.03 | 5.07 |
| Reported gestational age | DNAme ~ Cohort + Sex + Row + GA + ε | 21.18 | 4.51 |
| Epigenetic gestational age (RRPC) | DNAme ~ Cohort + Sex + Row + RRPC + ε | 21.99 | 4.58 |
| Ancestry | DNAme ~ Cohort + Sex + Row + Ancestry + ε | 19.26 | 4.08 |
| Cell Types | DNAme ~ Cohort + Sex + Row + Cell Types + ε | 12.89 | 3.23 |
| Reported gestational age, ancestry, and cell types | DNAme ~ Cohort + Sex + Row + GA + Ancestry + Cell Types + ε | 12.84 | 2.54 |
| Epigenetic gestational age, ancestry, and cell types | DNAme ~ Cohort + Sex + Row + RRPC + Ancestry + Cell Types + ε | 13.51 | 2.76 |
